# Mobility Analysis of the Lumbar Spine with a Dynamic Spine-Correction Device

**DOI:** 10.3390/s23041940

**Published:** 2023-02-09

**Authors:** Wojciech Kaczmarek, Łukasz Pulik, Paweł Łęgosz, Krzysztof Mucha

**Affiliations:** 1 Bio.morph, ul. Walecznych 61, 03-920 Warsaw, Poland; 2 Department of Orthopedics and Traumatology, Medical University of Warsaw, 02-005 Warsaw, Poland; 3 Department of Immunology, Transplantology and Internal Diseases, Medical University of Warsaw, 02-006 Warsaw, Poland

**Keywords:** angular mobility, dynamic spine correction, exercise therapy, magnetic strip, inertial sensors, low-back pain, neuromuscular reeducation, rehabilitation research, spinal curvatures

## Abstract

According to data, 60–70% of the world’s population experience low-back pain (LBP) at least once during their lifetime, often at a young or middle age. Those affected are at risk of having worse quality of life, more missed days at work, and higher medical care costs. We present a new rehabilitation method that helps collect and analyze data on an ongoing basis and offers a more personalized therapeutic approach. This method involves assessing lumbar spine rotation (L1–L5) during torso movement using an innovative dynamic spine correction (DSC) device designed for postural neuromuscular reeducation in LBP. Spinal mobility was tested in 54 patients (aged 18 to 40 years) without LBP. Measurements were made with 12-bit rotary position sensors (AS5304) of the DSC device. During exercise, the mean lumbar spine rotation to the right was greater (4.78° ± 2.24°) than that to the left (2.99° ± 1.44°; *p* < 0.001). Similarly, the maximum rotation to the right was greater (11.35° ± 3.33°) than that to the left (7.42° ± 1.44°; *p* < 0.0001). The measurements obtained in the study can serve as a reference for future therapeutic use of the device.

## 1. Introduction

An estimated 7% of all visits to a primary care physician are due exclusively to low-back pain (LBP) [1]. LBP is also the most common musculoskeletal complaint that physiotherapists deal with [2]. Most people (60–70%) experience at least one episode of LBP at some point in their life [3]. As a result, those affected need to take painkillers or undergo rehabilitation, and in severe cases require surgery. The prevalence of LBP is estimated to range from 1.5% to 20%, which makes the condition a significant burden for patients and a challenge for the entire health-care system [4]. Global Burden of Disease studies showed LBP to be the 13th-leading cause of disability-adjusted life years (DALYs) in 1990 and the 9th in 2019 [4]. These data support the hypothesis that LBP is an increasingly prevalent condition that affects a large proportion of the working population [5].

Treatment options can be divided into conservative and surgical (the latter reserved for patients with specific pathologies, for example, spinal disk herniation). Pain complaints can be addressed with a unimodal or multimodal approach. The effectiveness of unimodal strategies is currently being questioned, and experts advocate a multidisciplinary approach that combines rehabilitation with other treatment modalities [6,7,8].

Medical treatment includes acetaminophen, nonsteroidal anti-inflammatory drugs (NSAIDs), muscle relaxants, opioids, and antidepressants. NSAIDs are very often prescribed as the first-line therapy [9]. It should also be assumed that a significant proportion of patients self-medicate with over-the-counter (OTC) NSAIDs [10]. This drug group is included in most clinical guidelines [11]. However, NSAIDs are well known for their adverse effects, which include gastrointestinal, cardiovascular, and hypersensitivity reactions [12,13]. Furthermore, a recent Cochrane meta-analysis indicated that NSAIDs have very limited efficacy in reducing LBP when compared with a placebo. Medical treatment temporarily improves symptoms, but does not address the cause of LBP [14].

One commonly accepted and safe method for the therapeutic management of LBP is a combination of rehabilitation and exercises [14]. Physiotherapy for the treatment of LBP includes muscle-strengthening and aerobic exercises, manual manipulation, and device-based therapies. Manual and device-guided physical therapies are believed to be relatively effective in LBP compared with other methods of conservative treatment [15,16]. Examples of device-guided therapy include an inflatable trunk muscle-strengthening cuff (RECORE) [16]; the David Spine Concept, which uses targeted movement and controlled loading [17], and the fixation, elongation, and derotation (FED) method [18]. The main advantage of device-guided therapy is the repeatability of interventions. The quality of the procedure is independent of the disposition of the rehabilitator, whose fatigue increases with physical treatment of consecutive patients. The limitations of this treatment modality are the price and size of the device and the need to train the person performing physical therapy.

**Figure 1:**
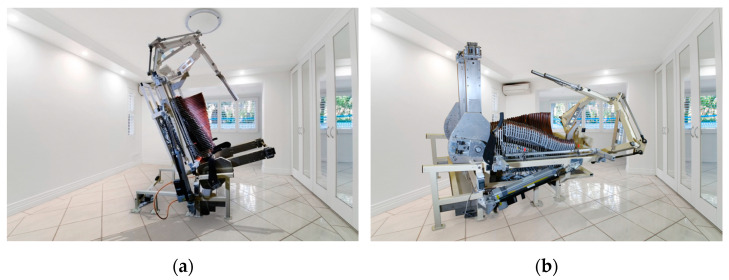
Dynamic spine correction (DSC) device; (**a**) the starting position and (**b**) a working position.

**Figure 2:**
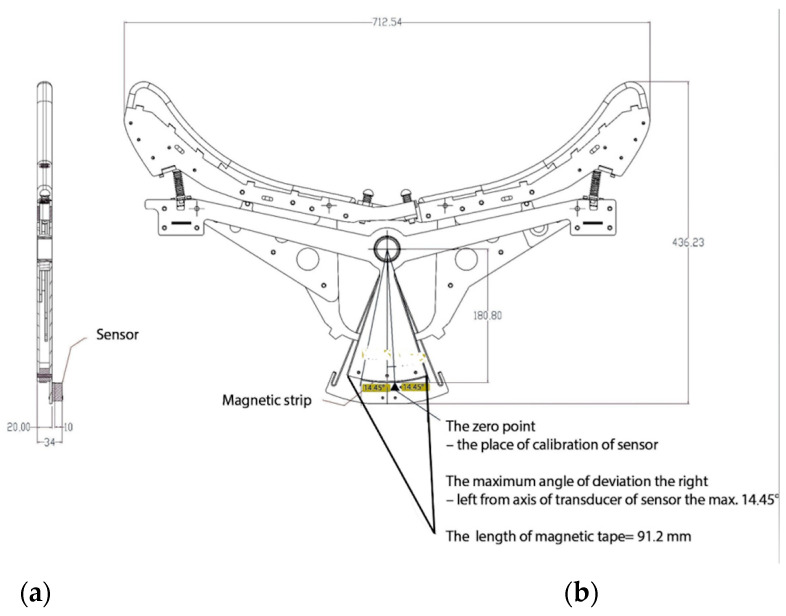
A single support segment, with its sensor and magnetic strip, viewed (**a**) from the side and (**b**) from the front. The base of the dynamic spine correction (DSC) device consists of moveable segments that adjust to and support the spine.

Recently, a dynamic spine correction (DSC) device has been developed for postural neuromuscular correction and reeducation (Figure 1a,b). The DSC device was designed and manufactured by Bio.morph under the European Union Innovative Economy Operational Programme 2007–2013 [19]. The working position for exercising on the DSC device is a neutral position of the spine, i.e., the natural curvatures are not flattened and the spine is not compressed. In this position, each spine segment is under the least possible tension, articular surfaces bear the least load, and muscles, ligaments, and articular capsules are relaxed. It is worth noting that lying on one’s back, even on a flat surface, cannot be considered a neutral position due to the physiological curvatures of the spine. Lying flat subjects individual spinal segments to excessive bending forces [20]. To ensure a neutral spine position, the base of the DSC device is made up of 33 movable segments that adjust to and support the entire length of the spine (Figure 2).

All exercises are performed with slight gravitational traction, resulting from the slanted orientation of the patient’s body relative to the ground. In this position, spinal movements are forced by synchronized flexion and extension of the upper and lower extremities of the patient. The most appropriate spine support distinguishes DSC from other device-based therapies.

This DSC device-guided therapy engages almost all muscle groups to restore joint mobility of the spine with simultaneous muscle training [19]. The DSC device is equipped with rotation sensors that continuously collect and analyze data on each spinal segment to determine the patient’s rehabilitation process.

In this paper, we present an analysis of lumbar spine (L1–L5) rotational mobility based on the measurements collected with a device for dynamic postural neuromuscular correction and reeducation. This study was carried out in patients without LBP to standardize the measurements obtained with the device’s built-in sensors. Our aim was to demonstrate the suitable operational functionality data collection of the system.

## 2. Materials and Methods

### 2.1. Study Design and Setting

Our study aimed to assess lumbar spine (L1–L5) rotation during movements of the torso on an innovative DSC device designed for postural neuromuscular reeducation in LBP.

This was a prospective observational study conducted at a private physiotherapy lab, Gabinet Fizjoterapii RC, in Warsaw, Poland. The eligibility evaluation was carried out by a general practitioner and orthopedic surgeon at an outpatient orthopedic clinic. The recruitment and data collection period lasted from November 2019 to July 2020.

### 2.2. Patients

The eligibility criteria for study participation were good health, age between 18 and 40 years, and a lack of current spinal pain. Patients with previous injury or current musculoskeletal dysfunction were excluded from the study. Other exclusion criteria were comorbidities that could adversely affect spinal mobility, including chronic diseases, such as inflammatory joint disease, neurodegenerative disorders, or malignancy.

The study sample size was determined by literature analysis [21]. All patients provided informed consent to participate in the study.

### 2.3. Dynamic Spine-Correction Device

The subjects of this study underwent evaluation of the torsional mobility of the lumbar spine (L1–L5) on the DSC device. The DSC device was patented at the Patent Office of the Republic of Poland (P.408841) [22] as well as at the United States Patent Office (US9949884B2) [23]. The device meets the conditions specified in the European Council Directive 93/42/EEC of 1993 and Directive 2007/47/EC of the European Parliament of 2007 on the use of medical devices.

**Figure 3:**
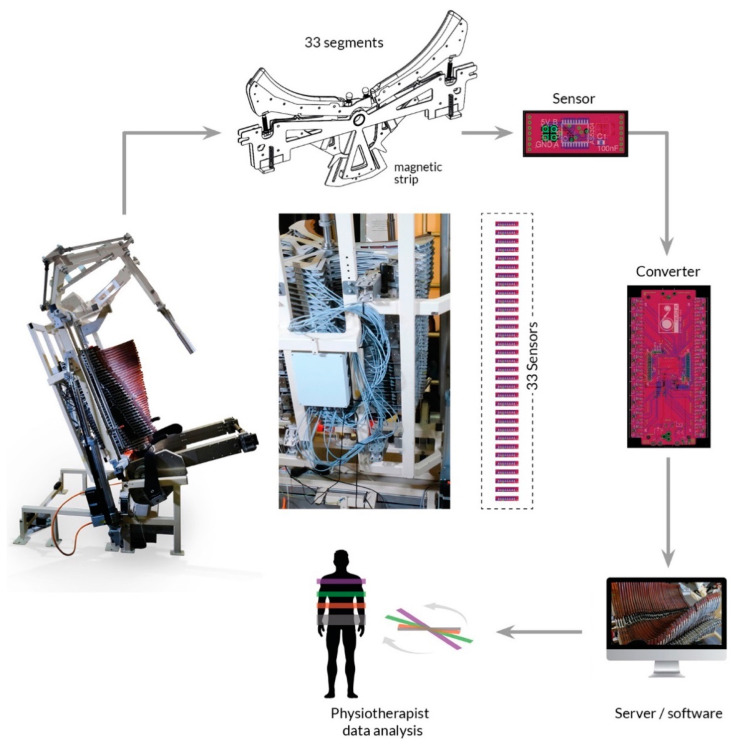
The dynamic spine correction (DSC) device has a “board,” which is a concave surface composed of a series of movable, curved elements (segments) that collectively support the patient’s back. The device includes a base and a mobile frame on which the board is mounted. The in-built measurement system includes a converter (hub), a server, and a monitor. The converter is connected to sensors that record the rotational positions of the individual segments in reference to the corresponding magnetic tape strips. The server provides feedback, which responds to the detected data, on the monitor.

During DSC kinesiotherapy, it is possible to record and monitor in real time the angular mobility of individual spinal segments. The structure of the DSC system is presented in Figure 3. The DSC device includes a base and a mobile frame on which the “board” is mounted. The “board,” which consists of 33 curved, movable elements (segments), forms a concave surface that supports the patient’s back (Figure 2). The patient is secured to the DSC device with a safety belt at the level of the anterior superior iliac spine (ASIS), which corresponds to the level of the S1 vertebra. The movable elements of the DSC device adjust to the spinal curvatures and to the length of the torso.

Information about the degree of rotation of each element (α’) is obtained from a magnetic tape (3M^®^) located on the next element by the sensor. The degree of rotation of each element is recorded relative to the position of the next adjacent element. The degree of rotation for a selected segment of the spine (α) can be calculated using the equation α = Σ α’n. The α calculated for the movement to right and left side (αR or αL) is a sum (Σ) of the rotation values of individual elements (α’). For the L1–L5 segment, the α values of the 5 final elements of the DSC device are added.

Each of the 12-bit rotary position sensors (AS5304) in the DSC device is situated 1 mm from the magnetic tape. The AS5304s are incremental position sensors for linear and rotary off-axis applications based on contactless magnetic sensor technology. Each sensor is located on a 1.6 mm-thick double-sided FR4 laminate circuit board. The AS5304 sensors obtain data from the magnetic tape using the Hall effect, which is a physical phenomenon involving the occurrence of a potential difference across a conductor in which an electric current flow when the conductor is in a magnetic field. The Hall effect is produced by the Lorentz force acting on charged particles moving in a magnetic field. The AS5304 sensor allows for high-speed (up to 20 m/s) contactless motion and position sensing. The resolution of the magnetic field sensor is 25 μm. From AS5304 sensors, the information is sent to a converter [24].

**Figure 4:**
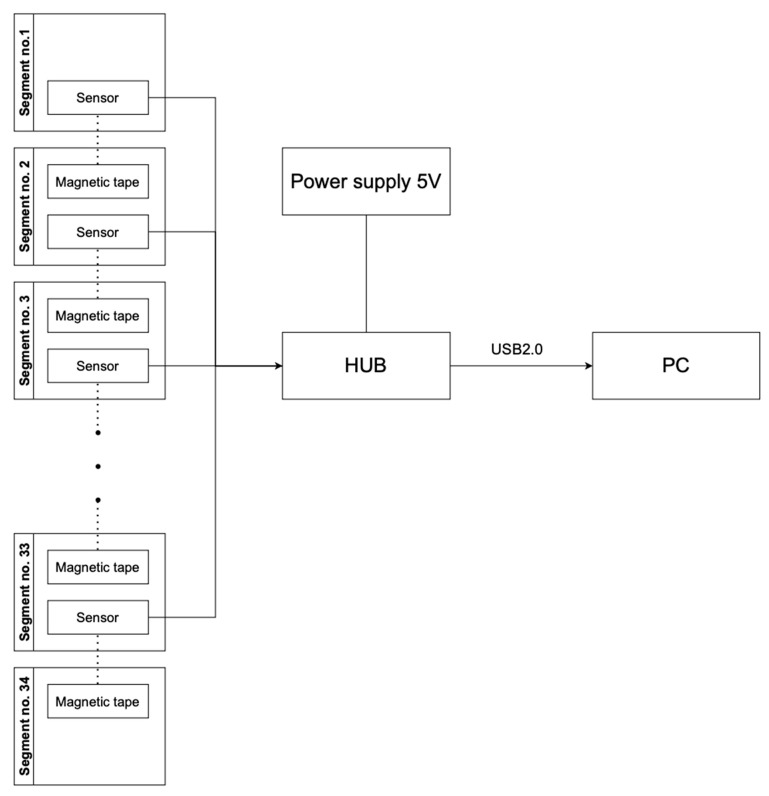
Information about the degree of rotation of each element is collected, via the Hall effect, by its sensor from the magnetic tape (3M^®^) strip located on the next adjacent element. The hub contains a power block, a USB communication block, sensor sockets, and a microcontroller. The information from the hub is analyzed via a Python-based application.

The converter (hub) contains a power block, a USB communication block, 20 sensor sockets, a STM32F407 microcontroller, and three differently colored LEDs that confirm that the converter is on. An expansion board, which is attached above the main board, expands the converter by 20 additional sensor sockets, bringing the total possible number of sensors to 40, 33 of which are used. The information from the microcontroller is sent to the server and analyzed by a Python-based application that provides output for the screen. The obtained data are expected to help evaluate the effectiveness and plan the course of physical therapy. A schematic drawing of the DSC device’s sensor system is presented in Figure 4.

DSC device performance was evaluated from a technological, functional, and utility-based perspective. Technologically, proper system functioning was ensured by a low-magnetic-field warning provided by any AS5304 sensor if the magnetic tape (3M^®^) was too far from the sensor. During the process of building the device, the sensors were tested by mechanically limiting their range of motion, their extreme ranges were compared with the sensor readings before integration on the device, and the repeatability of these measurements was checked. The hub’s differently colored LEDs confirm that it is on. The computer receives output from the sensors connected to the hub and the information on how many sensors are active. The Python-based application was tested with a manual testing approach to identify any bugs, errors, or anomalies [25]. From the functional perspective, the device was initially tested by volunteers recruited by one of the authors before the study started. To assure repeatability of the measurements, there is a start-up procedure that involves locking of the segments relative to each other in a uniform “zero” position, without rotation (before the patient is seated on the DSC device). When the patient is placed on the DSC the sensor locking is released and the measurements are always relative to the “zero” position. The device holds a Conformité Européenne (CE) certification, which required a review of technical documentation from the manufacturer on the safety and performance of the device.

### 2.4. Measurement Protocol

As it is necessary to apply force while using the device, patients must perform a warm-up to avoid injuries. The warm-up should continue without breaks and take the same amount of time—no less than 6 min—for each patient (further details on the adopted exercise methodology can be found in another article [26]). Subsequently, rotation of spinal segments is evaluated in a series of analyses.

**Figure 5:**
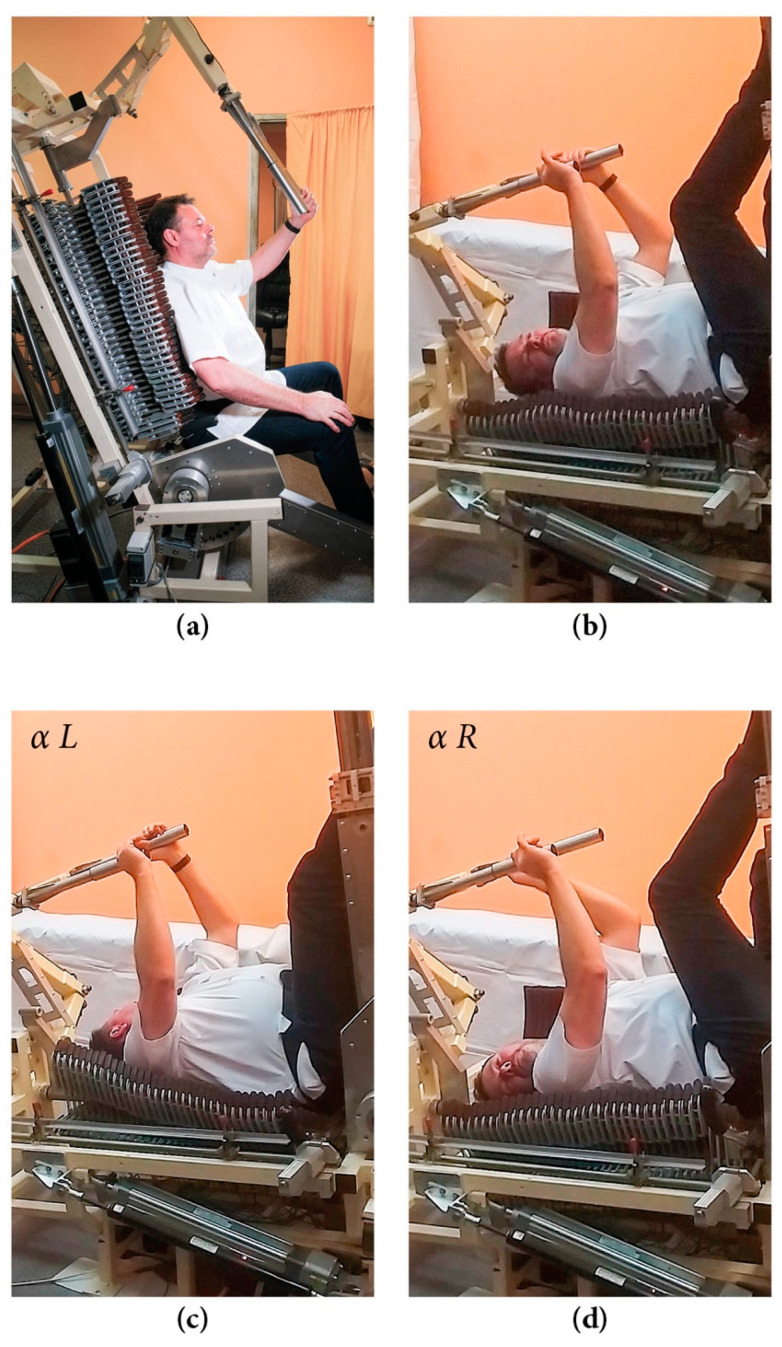
Dynamic spine correction (DSC) device positions. (**a**) Starting. the patient sits on the device in a vertical position and leans his back against the segments, which adjust to the spinal curvatures and support its entire length, while the safety belt at the level of the anterior superior iliac spines (ASIS) ensures the proper position of the patient. (**b**) Working–neutral: patient moves to the horizontal position, their hands and legs are placed on resistance handles, and they exercise in weak gravitational traction, resulting from a slanted positioning of the long axis of their trunk relative to the ground. (**c**) Rotation to the left (αL). (**d**) Rotation to the right (αR): the patient performs the exercise by alternately flexing and extending upper and lower limbs. The resistance handles allow the patient to perform torso rotational movements. In the long axis of the spine, the DSC has actuators that act as a spring and stretch the spine during the exercise. The αL and αR are measured by DSC sensors when the largest deviation is achieved from starting position.

Each assessed volunteer performed the assigned exercise in a uniform manner and continued for ten minutes. Measurements were collected automatically during the test. Each sensor automatically stored the data for each movement during the 10 min of exercise. The number of performed movements depended on the patients’ physical ability. The device has four possible settings: (a) starting, (b) working—neutral, (c) working—maximum rotation to the left (L), and (d) working—maximum rotation to the right (R) (Figure 5a–d). The video showing the patient exercising on the DSC while the analysis is performed can be accessed via the DSC device website [27].

In the starting position, the patient lies on his back and the movement is performed by a unilateral extension of an upper and lower extremity, so that the spine can rotate along the functional axis and extend (stretch out). The collection of all data from patient movements helps assess the mobility of individual spinal segments. The number of cycles during the 10 min workout depends on the physical capacity of the patient.

The data were saved and processed via a Python application to calculate the mean, minimum, and maximum degree of rotation. During a single cycle, the patient adopts the position of the maximum spine rotation to the left, the maximum rotation to the right, and the neutral working position. After finishing the 10 min series of cycles, the patient returns to the starting position.

Indications for stopping the exercise are shallow or wheezing breath, muscle cramps, evidence of impaired blood perfusion (confusion, ataxia, nausea, pallor, cyanosis, clammy skin), no changes in pulse on increasing effort, and palpitations.

DSC device calibration was repeated every time the system was disconnected from the power source and before every patient.

### 2.5. Statistical Analysis

Information about the patients’ sex, age, height, weight, body mass index (BMI), and hand dominance was recorded in a data collection form. The mean αR to right and αL to left were calculated for all cycles during the 10 min exercise on the DSC device. The highest and lowest degrees of rotation of all the cycles were also obtained for each side in every patient (see Section 2.3).

The normality of data was analyzed with the Shapiro–Wilk test. The test was used to assess continuous variables, such as age, height, weight, BMI, and lumbar spine rotation values (αR, αL). A paired *t*-test was used for continuous variables, with the assumption of normal distribution. For variables with abnormal distribution, the null hypothesis of equal samples was tested with the nonparametric Mann–Whitney U test. The accuracy of the DSC device was not measured, as it was not the purpose of the study to compare it to another device to serve as a reference value, while the precision was measured as the standard deviation, which is given in the Section 3. Statistical significance was set at <0.05. The software used for statistical analyses was Statistica 13.3 by TIBCO Software Inc. All data were collected on a central computer server. Sensitive personal data, based on which it would be possible to identify the patients in the future, were neither entered nor stored. Only the data necessary for the evaluation, i.e., the purpose of this project, such as patient age, sex, weight, and height, and the duration of the assessment, were recorded.

## 3. Results

### 3.1. Patients

In sum, 55 healthy subjects, 25 women (45%) and 30 men (55%), aged between 18 and 39 years were enrolled in the study. There were sex-related differences in the patients’ BMI, height, and weight. The men were taller (178.70 ± 7.33 cm) than the women (164.05 ± 9.64 cm). Body weight was also higher in men (89.80 ± 18.99 kg) than in women (65.00 ± 8.09). A significant difference between men and women was found in terms of BMI (27.92 ± 4.33 vs. 24.14 ± 2.66, respectively). No evidence of a sex-dependent difference in age was found between the groups. All patients were right-handed. Data on the means and standard deviations for the continuous variables among patient characteristics are presented in Table 1.

### 3.2. Mobility Analysis of the Lumbar Spine

Lumbar spine (L1–L5) rotation was measured with the DSC device in 54 out of 55 patients. The data from the sensors were not recorded in the case of one woman due to a system error. The calculated spinal rotation to the right and to the left were the mean αR and αL values of all cycles during the 10 min exercise on the DSC device (Table 2). Mean rotation to the left (2.99° ± 1.44°) had a significantly lower value than that to the right (4.78° ± 2.24°) during the exercises on the DSC device (*p* = 0.0001). The highest recorded degree of rotation was also higher on the right side (11.35° ± 3.33°) than on the left side (7.42° ± 1.97°) (*p* < 0.0000). There was no difference in the lowest degree of rotation recorded on the left and right sides (Table 3).

The calculated angles of rotation were analyzed in terms of possible sex-related differences, yielding no significant difference for the calculated rotation measured with the DSC device during the 10 min exercise in the degree of rotation to the left and to the right (Table 4).

Table 5 compares the lowest and the highest degree of rotation to the left and to the right in men and women. The analysis did not reveal any significant sex-related differences between these parameters. Other patient-dependent factors, such as BMI, height, and weight, had no effect on the assessed parameters either.

## 4. Discussion

Given the substantial number of patients who report back pain, most often in the lumbar region, worldwide, the search for new technologies to improve the effectiveness of rehabilitation is well justified [5]. As mentioned above, device-guided physical therapy has the advantage of offering a level of repeatability and precision that no human can provide [19]. Recently, robot-assisted rehabilitation technologies, including body movement and position monitoring with appropriate measurement instruments, are being intensively developed [28,29].

Similarly, the DSC device has a unique system that makes it possible to record and analyze the progress of physiotherapy. The main advantage of this technology is the repeatability and high precision of physiotherapy resulting from the controlled manner of rehabilitation. The range-of-motion data are recorded in real time with the option of simultaneous tracking. Furthermore, automated recording technology provides objective data, which allows us to monitor changes between treatments and compare patients [19].

This study was conducted with an emphasis on patient safety, with the aim of establishing reference values for the future use of the DSC device. According to our results, the mean highest degrees of lumbar spine rotation to the right and to the left were 11.35° ± 3.33° and 7.42° ± 1.44°, respectively (*p* < 0.0001). The collected data on the degree values are consistent with those reported by Pearcy et al., who evaluated lumbar spine rotation in the standing position with a biplanar radiographic method. Those authors’ results showed approximately 2° of axial rotation at each lumbar intervertebral joint, with a total rotation of approximately 8°. An interesting aspect arising from that study is that the lordotic shape of the lumbar spine and the muscles controlling this spinal segment are the two main factors that affect its rotation [30]. Shin et al. investigated the axial rotation of the lumbar spine (L2–S1) using a 3D model of the spine created based on magnetic resonance and fluoroscopy images. The overall axial rotation was 11.4° ± 3.6° to the left and 11.9° ± 2.9° to the right [31]. However, in neither study was there a significant difference between the left and right sides. Our data show a different trend, which could be explained by the most likely greater force exerted by the right-handed patients toward the right side during the exercise on the DSC device, causing the resulting spinal rotation to the right to be more pronounced, as the range of motion depends on the force exerted by the patient. A study by Petersen et al. supports this explanation by demonstrating that the dominant hand in right-handed patients has 10% more grip strength than the nondominant hand. This phenomenon is called the “10% rule” and is used to set rehabilitation goals [32]. Moreover, Fortin et al. have shown handedness to be associated with a corresponding asymmetry of trunk muscles [33]. Significant differences in electromyography and torque were also found between the dominant and nondominant leg in a study conducted by Valderrabano et al. [34]. These results provide support for our explanation of the higher values of both the mean and maximum rotation to the right side on the DSC device.

The most important limitation of our study stems from the fact that the measurements were taken only once and measurement repeatability was not assessed. However, each patient performed an exercise continuously for 10 min, resulting in many cycles per patient, which increases measurement reliability. In our opinion, caution must be exercised in using these data as the basis for extrapolating the maximum biomechanical rotation of the spine because the highest recorded degree of rotation depended on the force used by the patient during the assessment. However, the obtained results are still valuable and will serve as a future reference for patients treated with the DSC device.

To date, research on spine-rehabilitation devices has focused primarily on mechanical devices without computerized motion analysis [19]. There have already been some attempts to analyze spinal movement with sensors to provide a more personalized approach to exercises [35,36,37]. The design and application of physical therapy or diagnostic devices, especially those that provide real-time analysis of patient rehabilitation data, may be a game changer in the treatment of LBP [38,39,40]. Orthopedic rehabilitation innovations, such as the use of electromagnetic sensors, robotic devices, mobile applications, and virtual reality (VR) technology, are gaining popularity [41]. To use such devices, we need to establish standardized reference values against which to compare patient results during treatment. The numerical values reflecting the torsional mobility of the spine are device-specific and must be tested for each new proposed method [42]. This is the first paper presenting this novel method of motion analysis with the use of a DSC device in a healthy population.

## 5. Conclusions

This is the first study with the use of a DSC device conducted in a group of patients with no history of back pain or disease. The study aimed to standardize the measurements collected with a DSC device. We also confirmed the proper operational functionality and data collection capability of the DSC device.

This project is a very innovative approach to physiotherapy of LBP. In the next phase, we plan to conduct a pilot study in a group of patients with lumbar spine pain. We will compare the results with the standardized measurements obtained from this study. The DSC system can also help evaluate other regions of the spine, and different patterns of back pain can be precisely evaluated in future research.

## 6. Patents

European Patent Office EP 2974709A1; US patent 9,949,884 [23]; Patent Office of the Republic of Poland (UPRP) (P.408841)—Pat.229766 [22].

## Figures and Tables

**Table 1:** Mean values and standard deviations of continuous variables for the study population (n = 55).

	Women	Men	All Patients	*p*-Value
Age (years)	30.56 ± 6.16	29.60 ± 6.33	30.04 ± 6.22	0.635
Height (cm)	164.05 ± 9.64	178.70 ± 7.33	172.04 ± 9.66	<0.001
Weight (kg)	65.00 ± 8.09	89.80 ± 18.99	78.53 ± 19.44	<0.001
BMI	24.14 ± 2.66	27.92 ± 4.33	26.20 ± 4.10	<0.001

BMI—body mass index.

**Table 2:** Rotation [degrees] of the lumbar spine (L1–L5) measured with a dynamic spine-correction device (n = 54).

	Mean	Median	Minimum	Maximum	Q25%	Q75%
Rotation ^1^ (R)	4.78° ± 2.24°	4.55°	1.37°	9.85°	3.01°	6.18°
Rotation ^1^ (L)	2.99° ± 1.44°	2.59°	0.79°	6.38°	1.98°	4.00°

R—right side; L—left side; ^1^ the mean αR and αL values of all cycles during 10 min exercise on the dynamic spinal correction device.

**Table 3:** The highest and lowest degree of rotation of the lumbar spine (L1–L5) measured with the dynamic spine-correction device (n = 54).

	Mean	Median	Minimum	Maximum	Q25%	Q75%
Highest degree of rotation ^1^ (R)	11.35° ± 3.33°	11.57°	4.30°	17.82°	9.16°	13.48°
Lowest degree of rotation ^2^ (R)	0.03° ± 0.02°	0.02°	0.01°	0.10°	0.01°	0.03°
Highest degree of rotation ^1^ (L)	7.42° ± 1.97°	7.36°	2.57°	10.95°	5.97°	8.85°
Lowest degree of rotation ^2^ (L)	0.02° ± 0.02°	0.02°	0.01°	0.10°	0.01°	0.02°

R—right side; L—left side; ^1^ αR and αL values of one cycle with the highest degree value; ^2^ αR and αL of one cycle with the lowest degree value.

**Table 4:** Rotation [degrees] of the lumbar spine (L1–L5) in men and women.

	Mean	Median	Minimum	Maximum	Q25%	Q75%
Rotation ^1^ (R)—men (n = 30)	4.29° ± 2.02°	4.00°	1.48°	8.79°	2.49°	5.84°
Rotation ^1^ (R)—women (n = 24)	5.39° ± 2.39°	5.26°	1.37°	9.85°	3.94°	6.69°
Rotation ^1^ (L)—men (n = 30)	3.01 ± 1.49°	2.57°	1.15°	6.38°	1.81°	4.00°
Rotation ^1^ (L)—women (n = 24)	2.97° ± 1.41°	2.65°	0.79°	6.00°	2.04°	4.05°

R—right side; L—left side; ^1^ Rotation value was the mean αR and αL of all cycles during 10 min exercise on the dynamic spine-correction device.

**Table 5:** The highest and lowest degree of lumbar spine (L1–L5) rotation in men (n = 30) and women (n = 24).

	Mean	Median	Minimum	Maximum	Q25%	Q75%
Highest degree of rotation ^1^ (R)—men	11.47° ± 3.28°	11.89°	5.20°	17.82°	9.16°	13.48°
Highest degree of rotation ^1^ (R)—women	11.21° ± 3.46°	11.22°	4.30°	17.30°	9.18°	13.67°
Lowest degree of rotation ^2^ (R)—men	0.03° ± 0.02°	0.02°	0.01°	0.10°	0.01°	0.03°
Lowest degree of rotation ^2^ (R)—women	0.02° ± 0.02°	0.01°	0.01°	0.09°	0.01°	0.04°
Highest degree of rotation ^1^ (L)—men	7.85° ± 1.89°	7.71°	4.74°	10.95°	6.44°	9.99°
Highest degree of rotation ^1^ (L)—women	6.88° ± 1.96°	7.16°	2.57°	10.26°	5.58°	8.15°
Lowest degree of rotation ^2^ (L)—men	0.02° ± 0.01°	0.01°	0.01°	0.06°	0.01°	0.02°
Lowest degree of rotation ^2^ (L)—women	0.02° ± 0.02°	0.02°	0.01°	0.10°	0.01°	0.03°

R—right side; L—left side; ^1^ αR and αL of one cycle with the highest degree of rotation; ^2^ αR and αL of one cycle with the lowest degree of rotation.

## Data Availability

Data supporting the reported results can be obtained upon request from the corresponding author.
